# Effects of modified lipoproteins on human trophoblast cells: a role in pre-eclampsia in pregnancies complicated by diabetes

**DOI:** 10.1136/bmjdrc-2020-001696

**Published:** 2021-01-27

**Authors:** Rebecca Helen McLeese, Jiawu Zhao, Dongxu Fu, Jeremy Y Yu, Derek P Brazil, Timothy J Lyons

**Affiliations:** 1 Division of Endocrinology, Medical University of South Carolina, Charleston, South Carolina, USA; 2 Wellcome-Wolfson Institute For Experimental Medicine School of Medicine, Dentistry and Biomedical Sciences, Queen’s University Belfast, Belfast, UK

**Keywords:** diabetes mellitus, type 1, lipoproteins, pregnancy outcome, placenta

## Abstract

**Introduction:**

Pre-eclampsia (PE) is increased ~4-fold by maternal diabetes. Elevated plasma antiangiogenic factors, soluble fms-like tyrosine kinase (sFLT-1) and soluble endoglin (sENG), precede PE onset. We investigated whether diabetes-related stresses, modified lipoproteins and elevated glucose enhance trophoblast sFLT-1 and sENG release and/or alter placental barrier function and whether oxidized low-density lipoprotein (Ox-LDL) is in placental tissue.

**Research design and methods:**

HTR8/SVneo cells were exposed to ‘heavily-oxidized, glycated’ LDL (HOG-LDL) versus native LDL (N-LDL) (10–200 mg protein/L) for 24 hours ±pretreatment with glucose (30 mmol/L, 72 hours). Concentrations of sFLT-1 and sENG in supernatants (by ELISA) and expressions of *sFLT-1-I13* and *sFLT-1-E15A* isoforms, *endoglin (ENG*) and *matrix metalloproteinase-14* (*MMP-14*; by RT-PCR) were quantified. For barrier studies, JAR cells were cultured in Transwell plates (12–14 days), then exposed to LDL. Transepithelial electrical resistance (TEER) was measured after 6, 12 and 24 hours. In placental sections from women with and without type 1 diabetes, immunostaining of apolipoprotein B100 (ApoB, a marker of LDL), Ox-LDL and lipoxidation product 4-hydroxynonenal was performed.

**Results:**

HOG-LDL (50 mg/L) increased sFLT-1 (2.7-fold, p<0.01) and sENG (6.4-fold, p<0.001) in supernatants versus N-LDL. HOG-LDL increased expression of *sFLT-1-I13* (twofold, p<0.05), *sFLT-1-E15A* (1.9-fold, p<0.05), *ENG* (1.6-fold, p<0.01) and *MMP-14* (1.8-fold, p<0.05) versus N-LDL. High glucose did not by itself alter sFLT-1 or sENG concentrations, but potentiated effects of HOG-LDL on sFLT-1 by 1.5-fold (p<0.05) and on sENG by 1.8-fold (p<0.01). HOG-LDL (200 mg/L) induced trophoblast barrier impairment, decreasing TEER at 6 hours (p*<*0.01), 12 hours (p*<*0.01) and 24 hours (p*<*0.05) versus N-LDL. Immunostaining of term placental samples from women both with and without diabetes revealed presence of intravillous modified lipoproteins.

**Conclusion:**

These findings may explain, in part, the high risk for PE in women with diabetes. The trophoblast culture model has potential for evaluating novel therapies targeting barrier dysfunction.

**Significance of this study**

**What is already known about this subject?**

- In pregnancy, maternal diabetes increases risk of pre-eclampsia (PE) fourfold, but mechanisms are not well understood.
- Barrier dysfunction is an early general feature of complications of diabetes, as previously illustrated in diabetic retinopathy: low-density lipoprotein (LDL) permeates the diabetic retina, and once extravasated, oxidation and glycation confer cytotoxicity. An analogous process might occur in the placenta.

**What are the new findings?**

- In cultured human trophoblasts, modified LDL diminished cell viability, increased release of soluble fms-like tyrosine kinase (sFLT-1) and soluble endoglin (sENG) and induced barrier dysfunction.
- Effects of modified LDL were amplified by high glucose concentrations, which alone had no effect.
- Intravillous modified LDL is present in human term placentae.

**How might these results change the focus of research or clinical practice?**

- The trophoblast culture model has potential for evaluating novel therapies targeting barrier dysfunction.
- Inhibition of trophoblast signaling pathways activated by combined effects of modified lipoproteins and elevated glucose may ameliorate risk for PE in women with diabetes.

## INTRODUCTION

Pre-eclampsia (PE) is a major cause of maternal and neonatal morbidity and mortality; it is ~4-fold more frequent in women with diabetes (~20% vs 5%).^1^ The pathogenesis of PE is poorly understood, but in the second trimester, elevated maternal plasma concentrations of antiangiogenic factors, soluble fms-like tyrosine kinase (sFLT-1) and soluble endoglin (sENG), are predictive.^2 3^ In women with type 1 diabetes, elevated sFLT-1 predicts PE as expected, but sENG appears to rise excessively in all women with diabetes, perhaps explaining their susceptibility.^4^ sFLT-1 is formed by alternative splicing^5^; the main splice variants are *sFLT-1-I13*, the originally identified form, and *sFLT-1-E15a*, the placenta-specific form^6^; both are upregulated prior to PE onset.^6 7^ sENG is a proteolytic cleavage product of transmembrane ENG; the mechanism for its enhanced release in PE is unclear but may be mediated by increased matrix metalloproteinase-14 (MMP-14) expression or activity.^8 9^

Other factors associated with vascular complications of diabetes, including hyperglycemia and dyslipidemia, may modulate PE.^10 11^ In a prospective study of pregnant type 1 diabetic women, we found that plasma low-density lipoprotein (LDL) was elevated early in pregnancy in those with subsequent PE.^11^ It is well established that extravasated oxidized LDL (Ox-LDL) in arterial intima mediates atherosclerosis. Likewise, breakdown of the blood retinal barriers permits analogous extravasation and oxidation of LDL^12^; we found extensive circumstantial evidence implicating these effects in retinal injury.^12–16^ One key lipoxidation product is 4-hydroxynonenal (4-HNE), which has potential to be a surrogate for Ox-LDL in cell culture work.

From these considerations, we postulate that placental lipoprotein extravasation and modification may operate as both cause and consequence of placental barrier dysfunction, thus promoting PE. Metabolic stresses (high/fluctuating glucose concentrations, free fatty acids, oxidative stress, osmotic stress) may contribute to gradual, progressive barrier breakdown, allowing plasma constituents, including lipoproteins, to invade normally inaccessible tissue compartments. Previous studies suggest placental barrier dysfunction in diabetes.^17–19^ We hypothesize that exposure of placental trophoblasts to modified LDL and/or high glucose concentrations could compromise the maternal–fetal barrier and modulate development of PE.

The aims of this study were: (1) to investigate sFLT-1 and sENG release and expression from invasive trophoblast cells, which are readily exposed to ‘heavily oxidized, glycated LDL’ (HOG-LDL) and/or elevated glucose; (2) to study effects of these stresses on barrier function of trophoblast monolayer; and (3) to explore whether extravasated LDL is present in human placentae at term from women with and without diabetes.

## Research design and methods

### Human lipoprotein preparation

Native LDL (N-LDL) and HOG-LDL were prepared as previously described.^12^ Briefly, plasma was pooled from three or four healthy fasted volunteers who were taking neither antioxidant vitamins nor prescribed medications. N-LDL was isolated by sequential ultracentrifugation (350 000g, density (d)=1.019–1.063 g/mL). Glycated LDL (G-LDL) was prepared by incubation with glucose (50 mmol/L, 72 hours, 37°C) under antioxidant conditions (1 mmol/L diethylenetriamine pentaacetate, 270µmol/L EDTA). HOG-LDL was prepared by oxidizing G-LDL with 10 µM copper chloride (CuCl_2_) (24 hours, 37°C), followed by repeated dialysis against EDTA (24 hours, 4°C). LDL protein concentration was measured (Pierce BCA Assay, Thermo Fisher Scientific, Rockford, Illinois, USA). LDL fluorescence (ex 355 nm/em 460 nm) and absorbance (234 nm) were determined, and agarose gel electrophoresis performed (SAS-MX Lipoprotein gel, Helena Biosciences Europe, Gateshead, UK) to confirm modification. Preparations were confirmed as endotoxin negative (Limulus Amebocyte Lysate kit, Lonza, Allendale, New Jersey, USA), and cytotoxicity was determined as absent for N-LDL and present for HOG-LDL (cell counting kit-8 (CCK-8) assay, Dojindo Molecular Technologies, Rockville, Maryland, USA). LDL preparations were stored in dark under nitrogen (4°C). Preparations were used within 1 month and experiments were repeated using different LDL preparations.

### Human trophoblast HTR8/SVneo cell culture

The first trimester trophoblast cell line HTR8/SVneo was a gift from Professor Charles H Graham, Queen’s University, Ontario, Canada. Cells were seeded into six-well plates (3×10^5^ cells/well), maintained overnight in Roswell Park Memorial Institute (RPMI)-1640 medium (Thermo Fisher Scientific) supplemented with 10% fetal calf serum (FCS) (Thermo Fisher Scientific) (37°C, 5% CO_2_) and reached 70% confluence the next day. They were made quiescent by overnight exposure to serum-free medium (SFM), then incubated with N-LDL, HOG-LDL (10, 25, 50, 100 or 200 mg protein/L) or 4-HNE (Cayman Chemical, Ann Arbor, Michigan, USA) (5-40 µmol/L) in PBS for up to 24 hours. In separate experiments to address effects of elevated glucose, cells were seeded into six-well plates (1.5×10^5^ cells/well) and maintained overnight in RPMI medium with 10% FCS. The next day, relevant concentrations of glucose or mannitol (osmotic control) were spiked into the media. HTR8/SVneo cells did not survive 5 mmol/L D-glucose possibly due to the rapid glucose consumption over 48 hours; therefore, 11 mmol/L D-glucose in its standard culture medium was designated as the glucose control. For high glucose conditions, cells were exposed to 30 mmol/L D-glucose (ie, 11 mmol/L D-glucose and 19 mmol/L additional D-glucose or mannitol). After 48 hours, cells were serum-starved (24 hours) with no change in glucose/mannitol concentrations, then exposed to N-LDL versus HOG-LDL (50 mg LDL protein/L, 24 hours). Total treatment time in 11 mmol/L versus 30 mmol/L glucose was 96 hours. Each condition was studied in duplicate, and each experiment was performed in triplicate using different LDL preparations.

### Human trophoblast JAR cell culture

Since cytotrophoblast HTR8/SVneo cells do not form a monolayer in Transwell plates, JAR cells (ATCC, Manassas, Virginia, USA) were used for this purpose. They were cultured for 12–14 days in RPMI-1640 medium with 10% FCS (medium changed every 2 days) then for 24 hours in RPMI-1640 with 1% FCS (JAR cells do not tolerate SFM). N-LDL or HOG-LDL (200 mg protein/L) were then added to the top of the semipermeable membrane (representing the apical side of the trophoblast) or the bottom (representing the basal side of the trophoblast) for up to 24 hours. Each condition was studied in duplicate with experiments in triplicate using different LDL preparations.

### Cell viability assay

Cells were seeded into 96-well plates (2×10^4^ cells/well). After exposure to experimental conditions for 24 hours, cell viability was measured by CCK-8 (Dojindo Molecular Technologies) per manufacturer’s instructions. Briefly, CCK-8 utilizes a water-soluble tetrazolium salt WST-8. On reduction by dehydrogenases in cells, a yellow coloured formazan product is formed, which is proportional to the number of living cells. The CCK-8 results were consistent with the trypan blue exclusion assay in our studies (data not shown).

Measurement of sFLT-1 and sENG in Culture Supernatants sFLT-1 and sENG in supernatants was measured by Quantikine ELISA assays (R&D Systems, Minneapolis, Minnesota, USA) per manufacturer’s instructions. The interassay coefficients of variation (CVs) were 10% and 16%, respectively, and intra-assay CVs were 9% and 8%, respectively. Data were presented as ‘cell viability adjusted’, that is, the biomarker of interest (ng/L)/proportion of alive cells.

### Quantitative real-time PCR

RNA extraction (RNeasy Mini Kit; Qiagen, Valencia, California, USA) was followed by cDNA synthesis (Superscript III, Invitrogen, Carlsbad, California, USA). Semiquantitative real-time PCR was performed for the *sFLT-1* isoforms *sFLT-1-I13* and *sFLT-1-E15A*, *ENG* (endoglin) and *MMP-14*. mRNA levels were normalised to *ACTB* (ẞ-actin). Relative quantitative values were obtained (ΔΔCt method). Primer sequences included *sFLT-1-I13* forward: ACAATCAGAGGTGAGCACTGCAA, *sFLT-1-I13* reverse: TCCGAGCCTGAAAGTTAGCAA, *sFLT-1-E15A* forward: ACACAGTGGCCATCAGCAGTT, *sFLT-1-E15A* reverse: CCCGGCCATTTGTTATTGTTA; *ENG* forward: CAACAACCAAGGGCTGGGG, *ENG* reverse: TGGAGATGGGACGGGTATGC; *MMP-14* forward: CCTGCCTGCGTCCAT, *MMP-14* reverse: TCCAGGGACGCCTCATCA and *ACTB* forward: CAGTCGGTTGGAGCGAGCAT, *ACTB* reverse: GGATGGCAAGGGACTTCCTGTA.

### TEER measurements

Transepithelial electrical resistance (TEER) was measured using an epithelial voltmeter with EndOhm chamber (EVOM system, WPI Instruments, Sarasota, Florida, USA), per manufacturer’s instructions. The concentric electrodes were within the base of the chamber and the cap: a silver/silver chloride pellet in the centre plus an annular current electrode. An alternating current voltage (12.5 Hz square wave) was applied to the electrodes to avoid damage to both cells and electrodes. Cells were seeded on 12-well Transwell filters (12 mm diameter, 0.4 µm pore) (Thermo Fisher Scientific). The volume of media was 500 µL inside the Transwell insert and 1.5 mL outside. TEER was measured at room temperature prior to media change (every 48 hours). TEER levels were measured over a period of 2–3 weeks, until a plateau was reached.

### Immunohistochemistry in human placentae

Immunostaining of apolipoprotein B (ApoB), Ox-LDL and 4-HNE was performed in placental sections obtained at elective cesarean section beyond 32 weeks’ gestation. Placental tissues were collected following a standardized protocol to avoid bias.^20^ Biopsies were taken from each quadrant of the placenta (four sample areas from each placenta). Images presented here were representative of two sample areas per patient (two fields per sample area). Written informed consent was obtained from all participants. Participants included women with pregestational type 1 diabetes (n=3) and women without diabetes (n=4). In those with diabetes, HbA1c values at the first trimester were 9.1%, 12.1% and 6.5%, and at delivery were 6.8%, 6.7% and 5.6%, respectively. HbA1c was not routinely tested in women without diabetes. Maternal characteristics of the two groups are presented in online supplemental table 1.

10.1136/bmjdrc-2020-001696.supp1 Supplementary data

Formalin-fixed placental samples were dehydrated in a graded series of ethanol and embedded in paraffin. Serial sections (8 µm) were cut, mounted on glass slides and deparaffinized using xylene and a graded series of ethanol (5 min for each step). Placental sections were incubated with primary goat anti-human ApoB (1:100), rabbit anti-human Ox-LDL (1:1000) and mouse anti-4-HNE (1:100) (Abcam, Cambridge, Massachusetts, USA) (4°C, overnight), then incubated with secondary antibodies goat anti-rabbit (1:400), rabbit anti-goat (1:400), and goat anti-mouse, respectively (Vector Laboratories, Burlingame, California, USA) (37°C, 1 hour). Positive staining appeared brown after incubation with VECTASTAIN ABC reagent and DAB substrate. Sections were counterstained with hematoxylin. Control reactions omitting the primary antibodies did not reveal any staining. Sections were examined and images were captured using an Olympus microscope. Investigators were blinded to reduce bias in analyses.

### Statistical analysis

Data are presented as means±SD. Experiments were conducted in duplicate or triplicate, with each replicated ≥three times. Unpaired Student’s t*-*test or one-way analysis of variance followed by post hoc Bonferroni test were used as appropriate (Prism 7 software; GraphPad Software, La Jolla, California, USA). A p value of <0.05 was considered significant. To denote statistical significance, asterisks were used for differences between HOG-LDL and non-treated control and daggers for differences between N-LDL and HOG-LDL, unless stated otherwise.

## Results

### HOG-LDL decreased HTR8/SVneo cell viability

**Figure 1:**
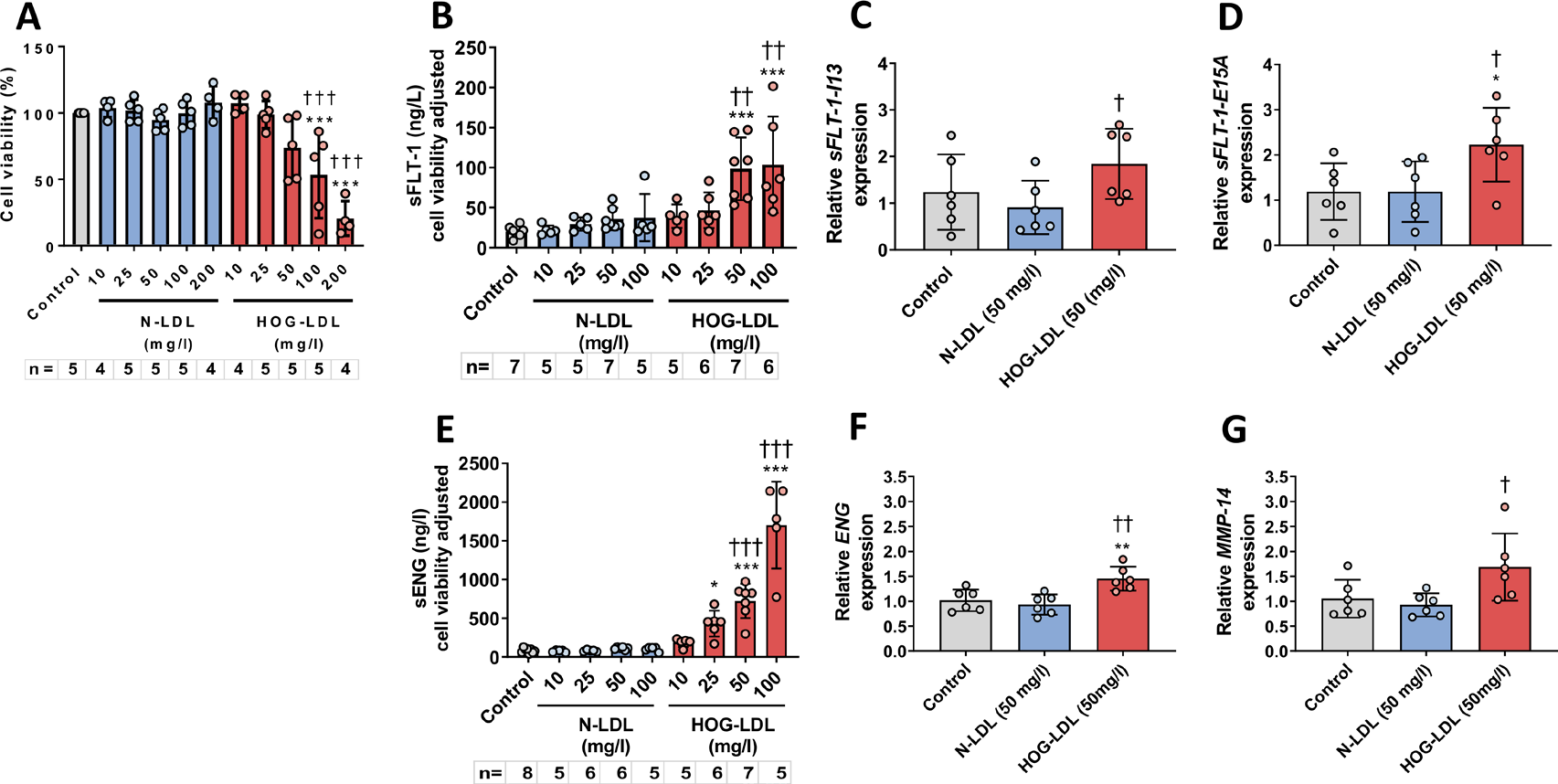
(A) Concentration-dependent changes of viability in HTR8/SVneo cells treated with N-LDL or HOG-LDL versus untreated control for 24 hours (***p<0.001 vs untreated control; ^†††^p<0.001 vs N-LDL at equivalent concentrations; one-way analysis of variance (ANOVA) with post hoc Bonferroni test). (B) HOG-LDL increased sFLT-1 release from semiconfluent HTR8/SVneo cells at 24 hours (***p<0.001 vs untreated control; ^††^p<0.01 vs N-LDL at equivalent concentrations; one-way ANOVA with post hoc Bonferroni test). (C) HOG-LDL (50 mg/L) increased *sFLT-1-I13* expression in HTR8/SVneo cells at 24 hours (p<0.05 vs N-LDL; unpaired Student’s t*-*test). (D) HOG-LDL (50 mg/L) increased *sFLT-1-E15A* expression in HTR8/SVneo cells at 24 hours (*p<0.05 vs untreated control; ^†^p<0.05 vs N-LDL; unpaired Student’s t*-*test). (E) HOG-LDL increased sENG release from semiconfluent HTR8/SVneo cells at 24 hours (*p<0.05, ***p<0.001 vs untreated control; ^†††^p<0.001 vs N-LDL at equivalent concentrations; one-way ANOVA with post hoc Bonferroni test). (F) HOG-LDL (50 mg/L) increased membrane *ENG* expression in HTR8/SVneo cells at 24 hours (**p<0.01 vs untreated control; ^††^p<0.01 vs N-LDL; unpaired Student’s t*-*test). (G) HOG-LDL (50 mg/L) increased *MMP-14* expression in HTR8/SVneo cells at 24 hours (^†^p<0.05 vs N-LDL; unpaired Student’s t*-*test). Data are presented as means±SD. HOG-LDL, heavily oxidized, glycated low-density lipoprotein; MMP-14, matrix metalloproteinase-14; N-LDL, native low-density lipoprotein; sENG, soluble endoglin; sFLT-1, soluble fms-like tyrosine kinase.

HOG-LDL (24 hours) decreased viability of HTR8/SVneo cells in a concentration-dependent manner, but N-LDL had no effect (figure 1A). JAR cells were more resistant, with significant cytotoxicity only when HOG-LDL concentration was ≥100 mg/L (online supplemental figure 1A).

### HOG-LDL induced sFLT-1 release and expression in HTR8/SVneo

As shown in figure 1B, sFLT-1 increased following 24 hours exposure to HOG-LDL (at 50 and 100 mg/L); N-LDL had no effect. At 50 mg/L HOG-LDL versus N-LDL, sFLT-1 concentrations were 99±39 ng/L versus 36±14 ng/L (p<0.001), respectively, while mRNA expression of *sFLT-1-I13* was increased 2.0-fold (n=6, p<0.05) and *sFLT-1-E15A* 1.9-fold (n=6, p<0.05) (figure 1C, D). sFLT-1 protein release by JAR cells could not be detected, and *sFLT-1-I13* and *sFLT-1-E15A* expression by JAR cells was low (data not shown).

### HOG-LDL induced release of sENG and enhanced expression of ENG in HTR8/SVneo

As shown in figure 1E, sENG in HTR8/SVneo increased following 24 hours exposure to HOG-LDL versus N-LDL (10–100 mg/L) in a concentration-dependent manner. At 50 mg/L HOG- versus N-LDL, sENG concentrations were 725±222 ng/L versus 113±12 ng/L (p<0.001), while mRNA expression of *ENG* was increased 1.6-fold (n=6, p<0.01) and *MMP-14* 1.8-fold (n=6, p<0.05) (figure 1F, G). In JAR cells, sENG release increased sixfold (n=4, p*<*0.05) following 24 hours exposure to HOG-LDL (50 mg/mL) (online supplemental figure 1B), but expression of *ENG* was low in these cells (data not shown), and expression of *MMP-14* did not change after exposure to HOG-LDL versus N-LDL (50 mg/L) (online supplemental figure 1C).

### 4-HNE only partially replicated effects of HOG-LDL on HTR8/SVneo

**Figure 2:**
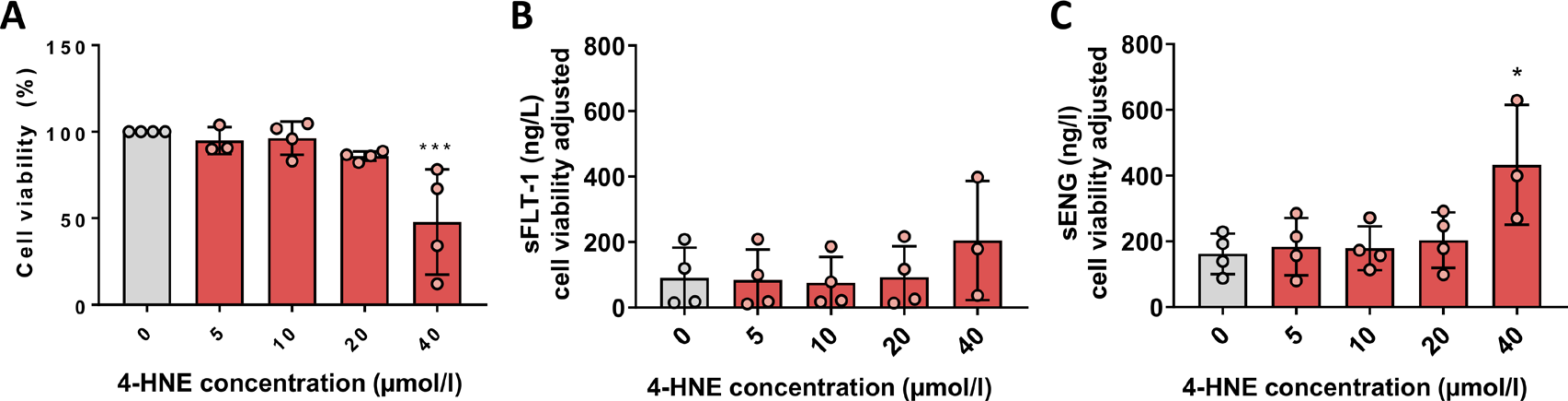
(A) 4-HNE at the highest concentration (40 µmol/L, 24 hours) decreased HTR8/SVneo cell viability (***p<0.001 vs untreated control; one-way analysis of variance (ANOVA) with post hoc Bonferroni test). (B) sFLT-1 release from HTR8/SVneo cells was not significantly affected by exposure to 4-HNE (5–40 µmol/L, 24 hours). (C) sENG release from HTR8/SVneo cells was increased by exposure to 4-HNE at the highest concentration (40 µmol/L, 24 hours) (*p<0.05 vs untreated control; one-way ANOVA with post hoc Bonferroni test). Data are presented as means±SD. 4-HNE, 4-hydroxynonenal; sENG, soluble endoglin; sFLT-1, soluble fms-like tyrosine kinase.

HTR8/SVneo cells were exposed to 4-HNE (5–40 µmol/L, 24 hours). Effects on cell viability differed from that of HOG-LDL: overall, 4-HNE was less toxic than expected from work with other cell types^15 20^; but at 40 µmol/L, viability decreased (n=4, p<0.01) (figure 2A). sFLT-1 and sENG were measured in cell supernatant following 4-HNE exposure (5–40 µmol/L, 24 hours): sENG but not sFLT-1 was increased 2.7-fold (n=3, p*<*0.05) (figure 2B, C).

### High glucose did not affect HTR8/SVneo viability or release of antiangiogenic factors

**Figure 3:**
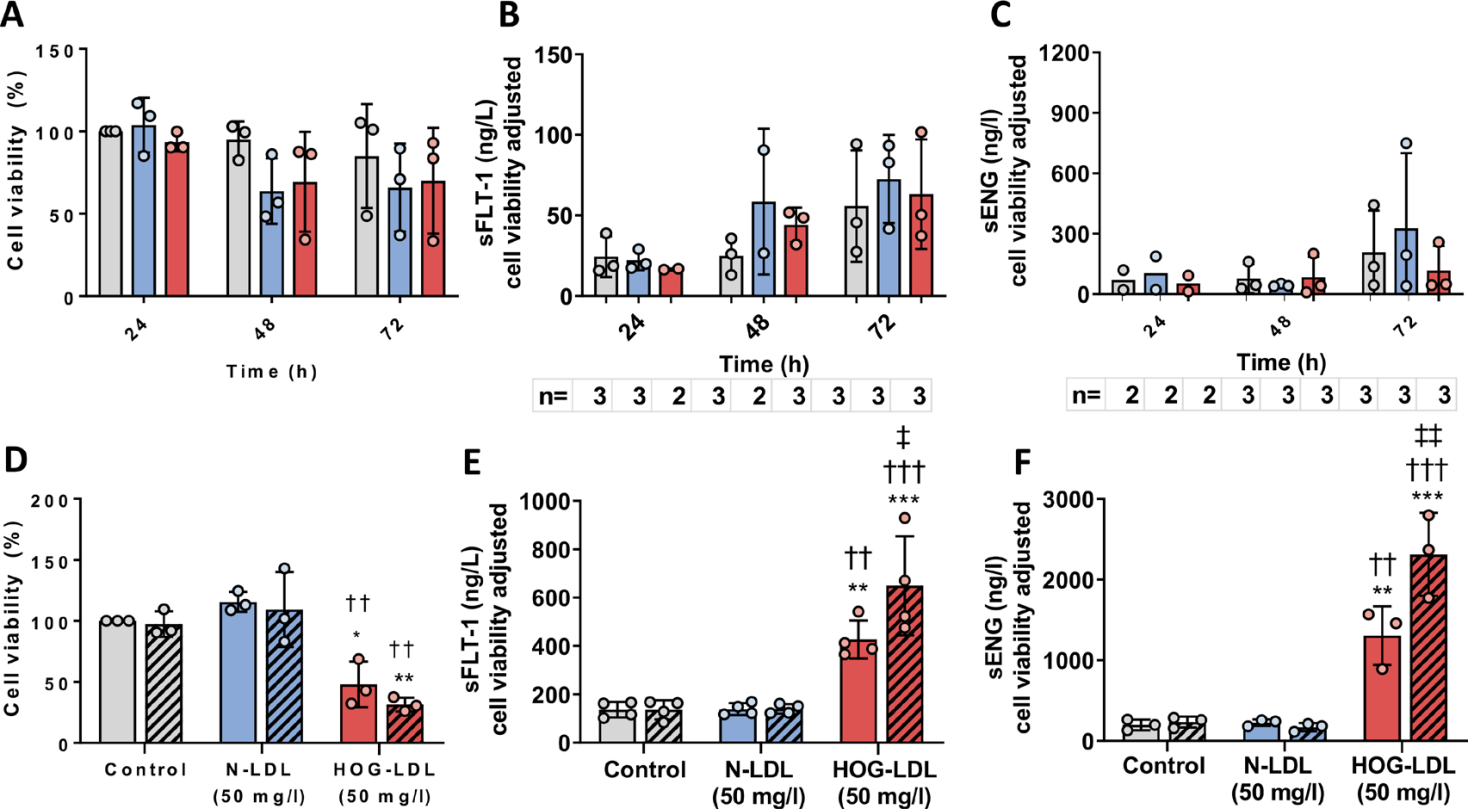
(A) Time-course (24–72 hours) viability of HTR8/SVneo cells exposed to 11 mmol/L D-glucose (glucose control), mannitol (osmotic control), versus 30 mmol/L D-glucose (high glucose). Release of (B) sFLT-1 and (C) sENG from HTR8/SVneo cells was not significantly affected by up to 72 hours exposure to high glucose (one-way analysis of variance (ANOVA) with post hoc Bonferroni test). Glucose control, grey bars; osmotic control, blue bars; high glucose, red bars. (D) HTR8/SVneo cell viability following pretreatment with high glucose, followed by N-LDL versus HOG-LDL exposure (*p<0.05, **p<0.01 vs untreated control at equivalent glucose concentrations; ^††^p<0.01 vs N-LDL at equivalent glucose concentrations; one-way ANOVA with post hoc Bonferroni test). Release of (E) sFLT-1 and (F) sENG from HTR8/SVneo cells was increased following HOG-LDL exposure, and this effect was amplified by high glucose pretreatment (**p<0.01, *** p<0.001 vs untreated control at equivalent glucose concentrations; †† p<0.01, ††† p<0.001 vs N-LDL at equivalent glucose concentrations; ‡ p<0.05, ‡‡ p<0.01 vs HOG-LDL in control glucose conditions; one-way ANOVA with post hoc Bonferroni test). Glucose control, filled bars; high glucose, patterned bars. Untreated control, grey bars; N-LDL, blue bars; HOG-LDL, red bars. Data are presented as means±SD. HOG-LDL, heavily oxidized, glycated low-density lipoprotein; MMP-14, matrix metalloproteinase-14; N-LDL, native low-density lipoprotein; sENG, soluble endoglin; sFLT-1, soluble fms-like tyrosine kinase.

As shown in figure 3A, neither high glucose (30 vs 11 mmol/L, 24–72 hours) nor mannitol affected HTR8/SVneo viability. Likewise, neither high glucose nor mannitol affected sFLT-1 or sENG concentrations in cell supernatants (figure 3B, C). Neither high glucose nor mannitol affected JAR viability or release of sENG in cell supernatants (online supplemental figure 2A, B).

### High glucose pretreatment enhanced the effects of HOG-LDL on HTR8/Svneo viability and release of antiangiogenic factors

High glucose pretreatment (30 vs 11 mmol/L, 72 hours) enhanced the effect of 50 mg/L HOG-LDL on cell viability (n=3, p<0.01) (figure 3D). Compared with N-LDL, HOG-LDL (50 mg/L) increased sFLT-1 and sENG release (figure 3E, F) and this was also amplified by pre-exposure to high glucose. Mannitol pretreatment did not affect HTR8/SVneo cell viability or release of antiangiogenic factors (data not shown). In contrast, JAR viability was similar following treatment with N-LDL and HOG-LDL (50 mg/L, 24 hours); however, high glucose pretreatment (30 vs 11 mmol/L, 72 hours) decreased viability with both N-LDL and HOG-LDL treatment (online supplemental figure 3A) and the effect of HOG-LDL on sENG was not amplified by high glucose (online supplemental figure 3B).

### HOG-LDL induced barrier breakdown in JAR cells

**Figure 4:**
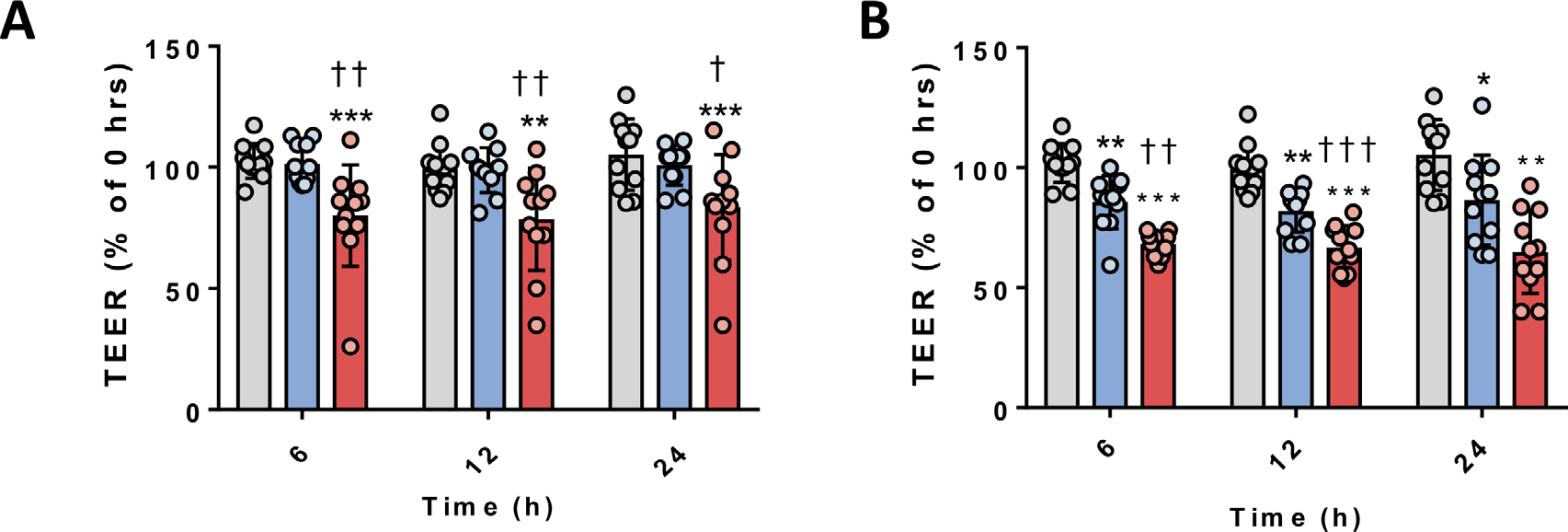
HOG-LDL decreased transepithelial electric resistance (TEER) in a trophoblast barrier model. JAR cells were cultured in Transwell plates for 12–14 days to form a monolayer, then treated with 1% fetal calf serum for 18 hours. Cells were exposed to N-LDL versus HOG-LDL (200 mg/L) for up to 24 hours. (A) TEER decreased following 6, 12 and 24 hours exposure to HOG-LDL added to the top chamber (apical side of the trophoblast) (**p<0.01, ***p<0.001 vs untreated control; ^†^p<0.05, ^††^p<0.01 vs N-LDL at equivalent time points; one-way analysis of variance (ANOVA) with post hoc Bonferroni test), or (B) TEER decreased following 6, 12 and 24 hours exposure to N-LDL or HOG-LDL added to the bottom chamber (basal side of the trophoblast) (*p<0.05, **p<0.01, ***p<0.001 vs untreated control; ^††^p<0.01, ^†††^p<0.001 vs N-LDL at equivalent time point; one-way ANOVA with post hoc Bonferroni test). Data are presented as means±SD percentages relative to the 1% FCS control at 0 hour. Untreated control, grey bars; N-LDL, blue bars; HOG-LDL, red bars. HOG-LDL, heavily oxidized, glycated low-density lipoprotein; N-LDL, native LDL.

HOG-LDL (200 mg/L) added to the top chamber (apical side of the JAR cell monolayer) caused a significant reduction of TEER at 6, 12 and 24 hours compared with N-LDL (n=11, p<0.01, p*<*0.01 and p*<*0.05, respectively) (figure 4A). HOG-LDL (200 mg/L) added to the bottom chamber (basal side of the JAR cell monolayer) caused a significant reduction of TEER at 6 and 12 hours, but surprisingly not at 24 hours, compared with N-LDL (n=11, p<0.01 and p*<*0.001, respectively) (figure 4B). Interestingly, N-LDL applied to the basal side also caused a reduction of TEER at all three time points (p<0.01, p<0.01 and p*<*0.05, respectively) but not as severe as that induced by HOG-LDL.

### Detection of ApoB, Ox-LDL and 4-HNE in placentae from women with and without diabetes

**Figure 5:**
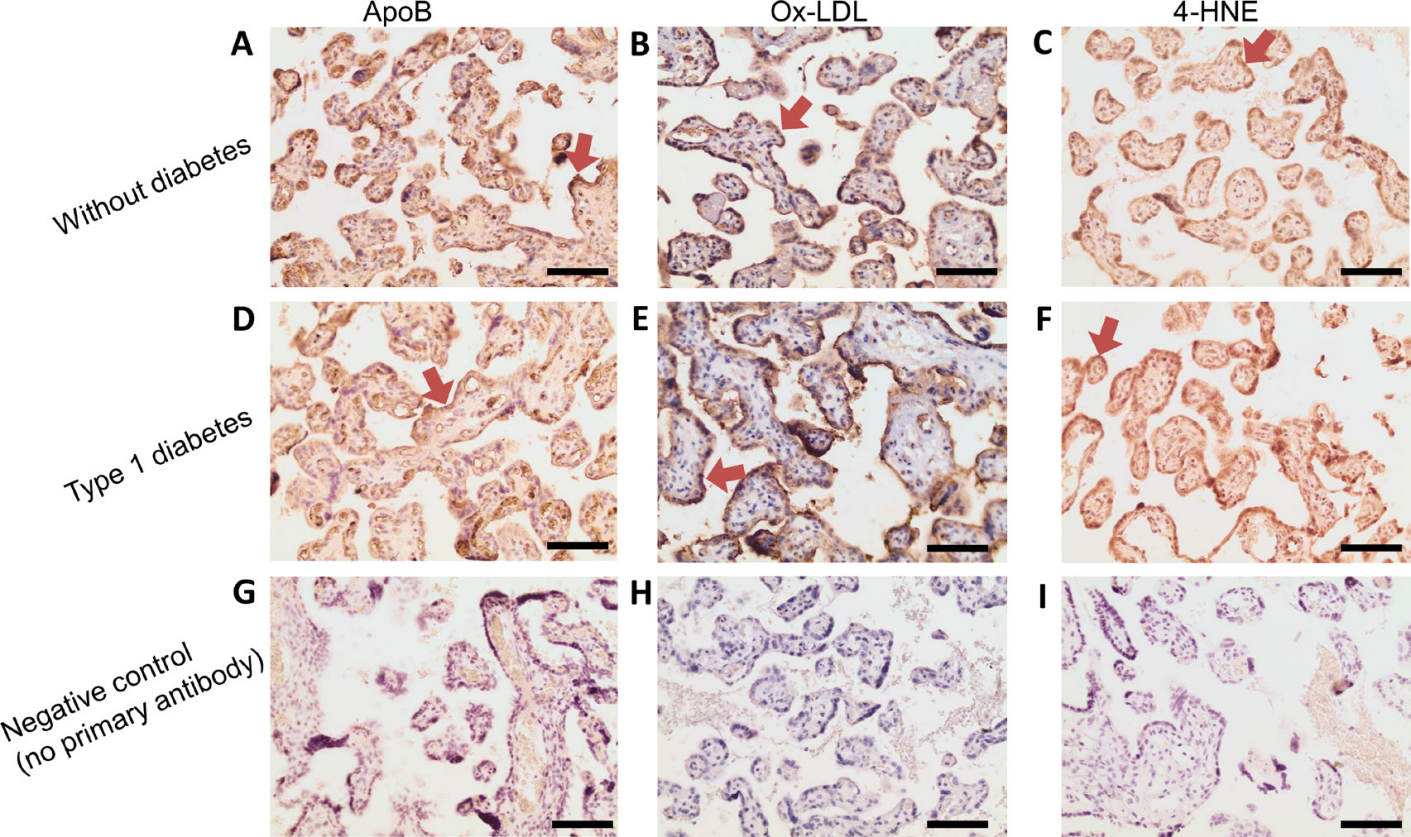
Presence of ApoB, Ox-LDL and 4-HNE in the human placenta. Representative placental images for (A) ApoB, (B) Ox-LDL and (C) 4-HNE in women without diabetes, and (D) ApoB, (E) Ox-LDL and (F) 4-HNE in women with type 1 diabetes. Positive staining was mainly in the syncytiotrophoblast layer (red arrows). Negative control for (G) ApoB, (H) Ox-LDL and (I) 4-HNE omitting the primary antibodies. Images are representative of negative control staining for all sections. Magnification: ×20. Scale bar=100 µm. ApoB, apolipoprotein B; 4-HNE, 4-hydroxynonenal; Ox-LDL, oxidized low-density lipoprotein.

To determine whether extravasated LDL that has undergone oxidation is present in human placental tissue, we performed immunohistochemistry to detect ApoB, Ox-LDL and 4-HNE in a small number of placental sections from women with either pregestational type 1 diabetes (n=3) or without diabetes (n=4). ApoB, Ox-LDL and 4-HNE were present in all placentae, regardless of diabetes status. The staining for all three was predominantly located in the syncytiotrophoblast layer (figure 5).

## Discussion

Maternal diabetes greatly increases risk for PE. To investigate mechanisms, we developed an in vitro model by exposing cultured immortalized human placental trophoblasts to modified lipoproteins, similar to those present in vascular tissues of patients with diabetes, and/or to high glucose. Our study is the first to demonstrate that in these cells modified LDL enhances release of two antiangiogenic factors sFLT-1 and sENG that are strongly implicated in PE, and that it induces barrier dysfunction. N-LDL had no effect, and interestingly, 4-HNE, a major fatty acid oxidation product known to replicate the effects of HOG-LDL in other cell types,^15 21^ had only modest effects. High glucose pretreatment amplified the effects of HOG-LDL, but by itself had no effect.

Supporting the relevance of these findings to PE, the concentrations of LDL used (10–200 mg/L) conservatively reflected those present in vivo. The highest concentration (200 mg protein/L) is equivalent to approximately 25% of plasma levels. Ox-LDL levels in atherosclerotic plaque are up to 70-fold higher than those in plasma^22^ and extravasated Ox-LDL tends to aggregate, so that local concentrations at sites of barrier leakage may be much higher than tissue averages. Using histochemistry, we demonstrated the presence of modified LDL in a small number of placental tissues, but further evidence is needed to conclude whether higher amounts are present in women with diabetes compared with women without diabetes and to assess any relationship with PE status. It is important to note that the placental samples studied were collected, of necessity, at the time of delivery by cesarean section. All showed the presence of Ox-LDL, but the rate of accumulation during gestation remains unknown. Possibly, women with diabetes accumulate modified lipids in placental tissues earlier than women without diabetes, yet all may eventually reach the same ‘saturation’ point. Enhanced accumulation earlier in pregnancy could potentially drive antiangiogenic effects throughout gestation in women with diabetes.

In the past, lipid abnormalities cross-sectionally associated with PE included high triglycerides,^23^ but prospectively, as reported in an earlier paper, we found no association with hypertriglyceridemia, but instead an association with first trimester LDL,^11^ suggesting a parallel with atherogenesis. Evidence exists that plasma Ox-LDL is elevated in women with PE, but its source is unclear.^24 25^

Limited studies have examined the presence of modified LDL in placental tissue. Such studies are challenging: it is important to control and standardize placental processing, to account for gestational age and to consider mode of delivery. An added problem concerns sample contamination by blood, since the placenta is highly vascular. Açıkgöz *et al*^26^ found that in PE, placental levels of Ox-LDL were lower than in healthy controls. This finding was against their (and our) hypothesis but their study may have been affected by some of the issues mentioned above.

In the current study, Ox-LDL was found in all human placentae analyzed, and our (at best semi-quantitative) initial findings show similar staining in placentae from women with and without diabetes. In agreement with our findings, Pecks *et al*^27^ reported Ox-LDL staining in trophoblast but to a lesser degree in villous stromal cells. The authors speculated that the process of oxidation resulted in accumulation of Ox-LDL particles at the apical placental surface, that is, the syncytiotrophoblast, reducing fetal cholesterol bioavailability. Importantly, results from term placentae may not be representative of what happens earlier in pregnancy, as discussed above. Pecks *et al*^27^ demonstrated increased accumulation of Ox-LDL within placentae of early-onset intrauterine growth restriction pregnancies compared with preterm control placentae. Basu *et al*^28^ conducted a study of placentae throughout normal gestation using tissues collected at termination and at term. They found that placental malondialdehyde concentrations were highest, and antioxidant defences lowest, early in gestation, concluding that placental oxidative stress declines as pregnancy advances. If so, the role of oxidized lipoproteins in pregnancies complicated by diabetes could be greatest early in gestation, that is, at times critical for successful placentation, when deficient angiogenesis may sow the seeds for later PE. These concepts have parallels in our own observations in women with type 1 diabetes: we observed associations of ‘adverse’ lipoprotein profiles early in gestation (first and second trimesters) with subsequent PE, but by the third trimester, these associations had disappeared.^11^

Few studies have examined the interaction of modified LDL with placental trophoblast cells.^29 30^ Bonet *et al*^30^ reported that Ox-LDL is toxic to trophoblasts consistent with our findings regarding HOG-LDL. The mechanisms involved are unclear and likely complex, however, recent studies have found a direct cytotoxic effect of sFLT-1.^31 32^ This provides a potential mechanism for the toxicity of Ox-LDL not only towards trophoblast but in other tissues where Ox-LDL is implicated: in arteries and in the diabetic retina.^12–16^

When considering the role of Ox-LDL particles within the placenta, it is important to appreciate that these particles do not represent a unique entity. In the current study, we found that 4-HNE-derived adducts (lipoxidation products formed within Ox-LDL), previously shown to be toxic to other cell types,^14 15 33^ had only modest effects on trophoblast cell viability and very little effect on the release of sFLT-1 and sENG. Previous studies provide evidence that treatment with oxysterols, a major group of components of modified LDL, increased *ENG* and sENG expression from trophoblast cells likely via increased expression of *MMP-14*.^9 34 35^ In the current study, there was a modest increase in *MMP-14* in response to HOG-LDL; this is consistent with the earlier finding that MMP-14 is the protease responsible for cleavage of ENG, increasing sENG release from trophoblast cells. Further work is needed to conclude if this is the case or if additional enzymes are also responsible.

Advanced glycation end products (AGEs) and lipoxidation end products (ALEs) are formed through free radical oxidation when proteins are exposed to reducing sugars and/or aldehyde-containing fatty acid fragments. In previous studies, exposure of HTR8/SVneo trophoblasts to AGEs/ALEs caused increased expression of *sFLT-1*.^36 37^ Plasma levels of AGE-ALE-modified LDL were elevated in patients with versus without diabetes,^38^ however, the role of AGEs in pregnancies complicated by diabetes and in the onset of PE is unclear. Li and Yang^39^ found that plasma AGEs in the second and third trimesters were significantly higher in women with gestational diabetes than those who were normoglycemic. In a cross-sectional study, Chekir *et al*^37^ found that women with PE had significantly higher serum AGEs than healthy pregnant and non-pregnant controls, while AGE-modified proteins, the AGE receptor, RAGE, and 4-HNE were elevated in pre-eclamptic versus healthy placentae. In our previous work, we did not find any significant difference in serum levels of two AGE species (Nᵋ-(carboxymethyl)lysine and hydroimidazolone) between the first and third trimester in women without diabetes versus women with type 1 diabetes, but decreased circulating soluble RAGE early in gestation (perhaps reflecting impaired ability to remove AGE products) was associated with later PE in type 1 diabetic women.^4^

Our initial results, although based on a small sample size, suggest that elevated glucose concentrations alone may not lead to an antiangiogenic response by trophoblasts, but co-existence of other factor(s), including the presence of modified lipoproteins whose formation is amplified by the presence of diabetes, may contribute. High glucose treatment (continuous or fluctuating) has previously been shown to increase *ENG*, sENG and sFLT-1 from trophoblast cell lines,^40–42^ however, in the present study, high glucose alone did not increase release of sFLT-1 or sENG from trophoblast cells.

There are limitations in our study. HTR8/SVneo and JAR cells responded differently to modified lipoprotein and high glucose exposure. While these immortalized cell lines maintain many of the morphological features of placental trophoblast,^43 44^ they are unlikely to be completely representative of normal physiology. HTR8/SVneo comprise two populations, trophoblast and stromal/mesenchymal cells,^45^ and whether our findings were complicated by the latter requires further investigation. The tumor origin of JAR cells^46^ may affect their proliferative responses. In addition, the glucose concentrations used in our culture experiments were, of necessity, higher than physiological levels. We report TEER measurements but recognise the need for additional evaluations of barrier structure and function. Preliminary fluorescein isothiocyanate (FITC-) dextran (10 kDa) permeability data suggested that leakage was unaffected by HOG-LDL, but electrical and leakage measures may reflect different properties of a cell monolayer, and other investigators have reported similar discrepancies.^47 48^ Future experiments should use a range of tracers with different molecular weights and Transwell membranes with different sized pores.

In summary, we present evidence that modified LDL may promote the development of PE, particularly in the context of maternal diabetes, through two distinct effects on trophoblasts: it enhances the release of antiangiogenic factors, and it impairs barrier function. Further studies are needed to investigate how trophoblasts release antiangiogenic factors in response to HOG-LDL and to elucidate whether and how modified LDL impairs placental barrier function. We also demonstrate that modified LDL is present in human placental villi at term, and even though, by semiquantitative measures, we found no difference in villous staining patterns of ApoB, Ox-LDL and 4-HNE in women with versus without diabetes, we show that elevated glucose levels amplify the effects of modified LDL. Future studies with a larger number of placentae with higher power images are needed. Improved understanding of the role of modified lipoproteins in promoting PE in diabetes may lead to new strategies to treat and prevent this common complication of pregnancy.
