# Supplementary material for: Effects of modified lipoproteins on human trophoblast cells: a role in pre-eclampsia in pregnancies complicated by diabetes
Source: BMJ Open Diabetes Res Care. 2021 Jan 27;9(1):e001696. doi: 10.1136/bmjdrc-2020-001696 (PMC7843297; doi:10.1136/bmjdrc-2020-001696)
Supplement: Supplementary data [file bmjdrc-2020-001696supp001.pdf]

Supplementary Table 1

Characteristic	No diabetes (n=4)	Type 1 diabetes (n=3)	p-value
Age of woman (years)	26.5 (4.8)	36.0 (6.3)	0.15
BMI at first trimester (kg/m^2)	29.5 (7.0)	27.3 (3.8)	0.86
HbA1c (%) at first trimester†	-	9.2 (2.8)	-
Gestational age at delivery (weeks)	38.7	36.8 (0.7)	0.06

Data are presented as mean ± SD or *n* (%) where appropriate. †HbA1c % unavailable for women without diabetes (n=4). Mann-Whitney U tests were used.

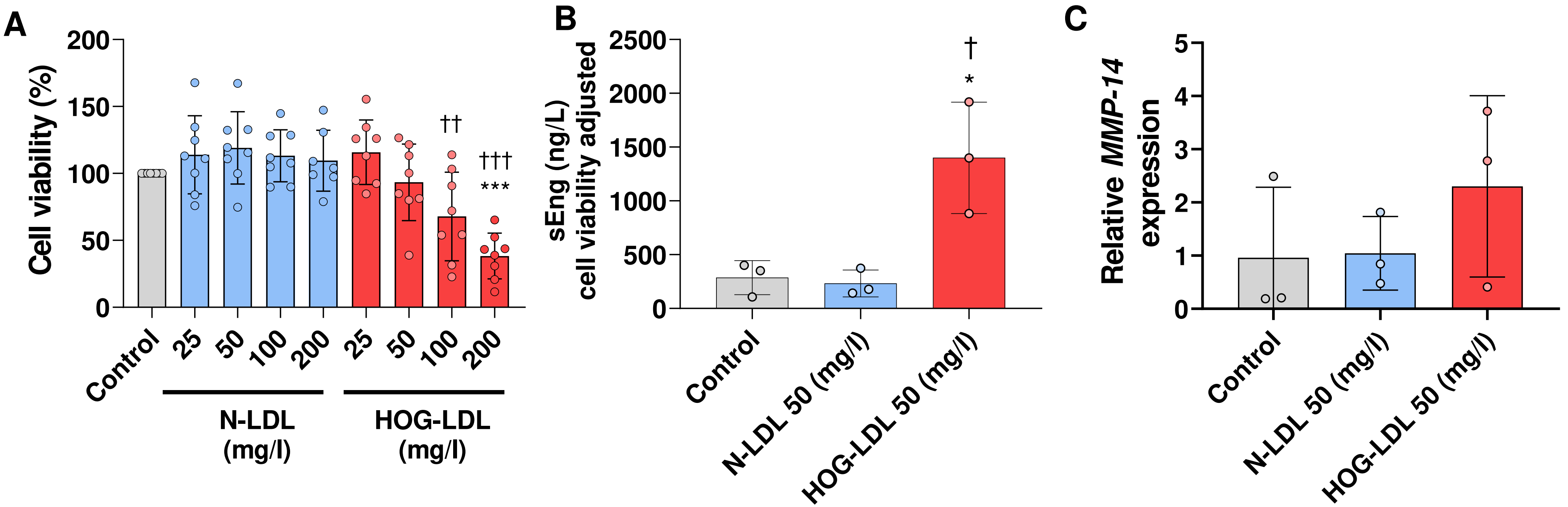

Supplementary Figure 1- HOG-LDL decreased JAR viability in a concentration dependent manner. sENG levels increased following HOG-LDL treatment, however, *MMP-14* expression remained unchanged. Untreated control, grey bars; N-LDL, blue bars; HOG-LDL, red bars. (A) Concentration-dependent changes of viability in JAR cells treated with N- or HOG-LDL vs. untreated control for 24h (***$p<0.001$ vs. untreated control; ††p<0.01, †††$p<0.001$ vs. N-LDL at equivalent concentrations; one-way ANOVA with post hoc Bonferroni test, n=8). (B) HOG-LDL (50mg/l) increased sENG release from semi-confluent JAR cells at 24h (*$p<0.05$, vs. untreated control; †$p<0.05$ vs. N-LDL (50mg/l); unpaired student *t*-test, n=3). (C) *MMP-14* expression in JAR cells was similar following N-LDL and HOG-LDL treatment at 24h (unpaired student *t*-test, n=3). Data are presented as means ± SD.

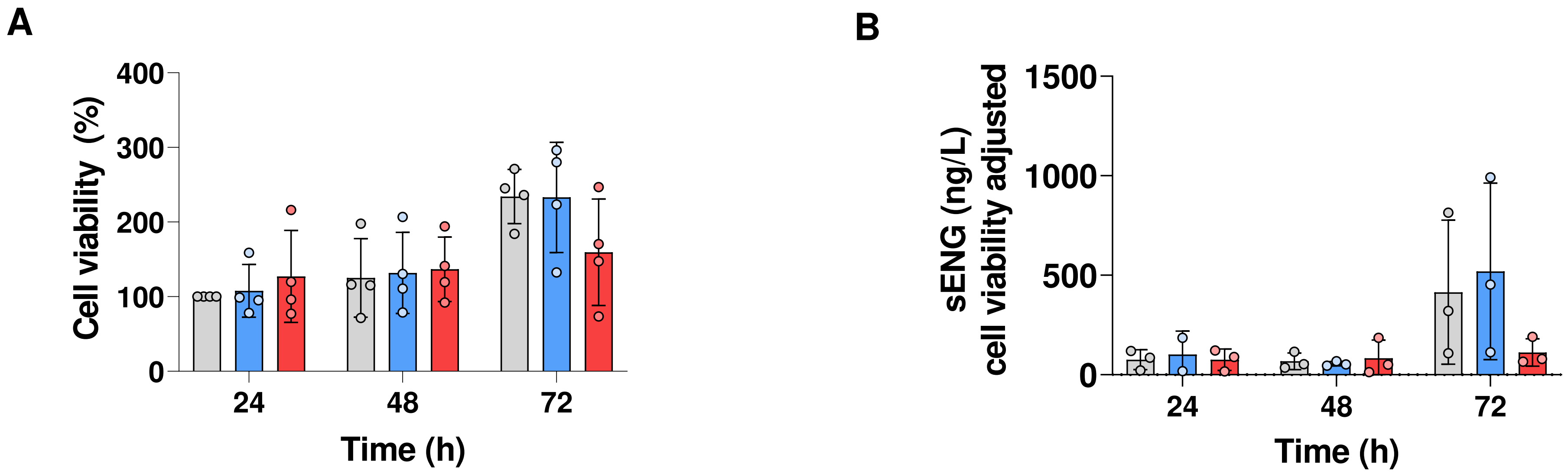

Supplementary Figure 2- Neither high glucose (30mmol/l) nor mannitol affected JAR viability or release of sENG. Glucose control, grey bars; osmotic control, blue bars; high glucose, red bars. (A) Time-course (24-72h) viability of JAR cells exposed to 11mmol/l D-glucose (glucose control), mannitol (osmotic control), vs. 30mmol/l D-glucose (high glucose). Release of (B) sENG from JAR cells was not significantly affected by up to 72h exposure to high glucose (one-way ANOVA with post hoc Bonferroni test). Data are presented as means ± SD.

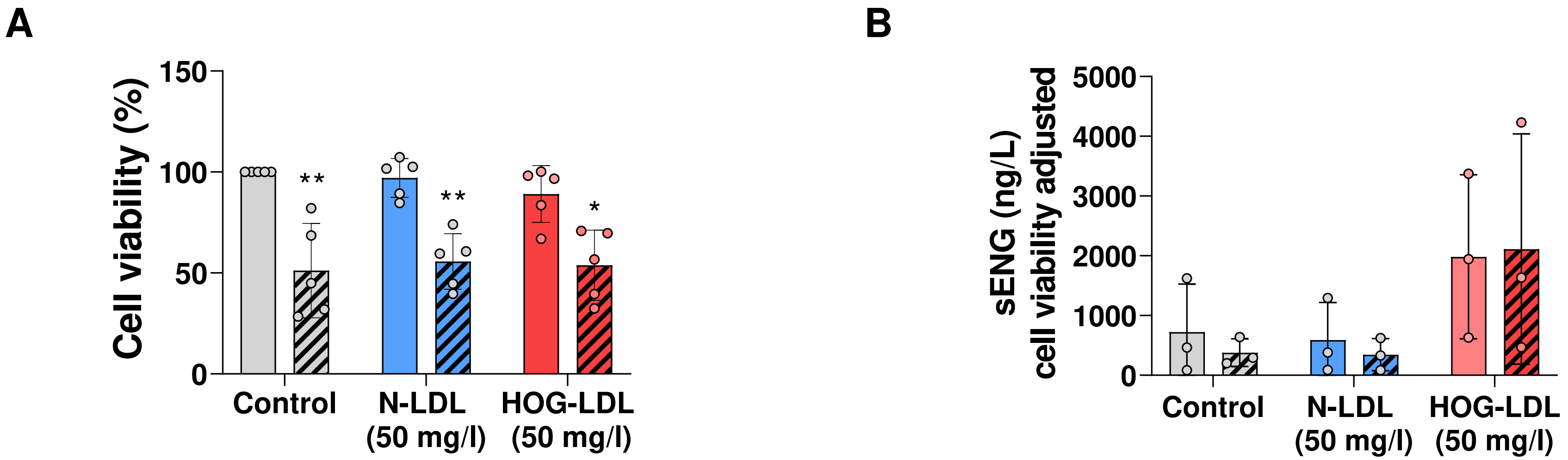

Supplementary Figure 3- High glucose pre-treatment conditions decreased JAR cell viability. The effect of HOG-LDL on sENG was not amplified by high glucose. Glucose control, filled bars; high glucose, patterned bars. Untreated control, grey bars; N-LDL, blue bars; HOG-LDL, red bars. (A) JAR cell viability following pre-treatment with high glucose, followed by N- vs. HOG-LDL exposure (*p<0.05, **p<0.01 vs. control glucose; one-way ANOVA with post hoc Bonferroni test). (B) Release of sEng into culture medium by JAR cells following pre-treatment (72hrs) with 11mmol/l D-glucose vs. 30mmol/l D-glucose , and treatment (24 hrs) with N-LDL vs. HOG-LDL (50 mg/l) (one-way ANOVA with post hoc Bonferroni test). Data are presented as means ± SD.
